# Moderate-to-Severe Depression Adversely Affects Lung Function in Chinese College Students

**DOI:** 10.3389/fpsyg.2020.00652

**Published:** 2020-04-15

**Authors:** Liya Guo, Jianhua Cao, Peng Cheng, Dongzhe Shi, Bing Cao, Guang Yang, Siyu Liang, Nan Su, Miao Yu, Chaowei Zhang, Rui Liang, Yaru Wang, Cuijin Bai, Chang Chen, Zhongyu Ren

**Affiliations:** ^1^College of Physical Education, Key Laboratory of Physical Fitness Evaluation and Motor Function Monitoring, General Administration of Sport of China, Institute of Sports Science, Southwest University, Chongqing, China; ^2^College of Physical Education, Chongqing Nursing Vocational College, Chongqing, China; ^3^Xinhua College, Sun Yat-sen University, Guangzhou, China; ^4^Key Laboratory of Cognition and Personality, Faculty of Psychology, Ministry of Education, Southwest University, Chongqing, China; ^5^School of Physical Education, Chinese Center of Exercise Epidemiology, Northeast Normal University, Changchun, China

**Keywords:** depression, depressive symptoms, lung function, forced vital capacity, chronic obstructive pulmonary disease

## Abstract

Depression is known to be correlated with increased risk for chronic obstructive pulmonary disease (COPD) in middle-aged and older adults, but there is scarce evidence regarding its association with lung function among healthy adults. Thus, we aimed to assess this association by measuring the lung function and depression severity in Chinese college students. This cross-sectional study was conducted among 3,891 college students aged 16–24 years. Lung function was assessed by measuring the forced vital capacity (FVC) using a spirometer, and depression severity was evaluated using the 20-item Zung self-rating depression scale (SDS), with SDS scores of ≥40 and ≥45 indicating mild and moderate-to-severe depression, respectively. After adjusting for potential confounders, the geometric means of the FVC levels for the normal, mild depression, and moderate-to-severe depression groups were 3,446.1 (95% confidence interval [CI]: 3,418.6–3,470.3), 3,415.2 (95% CI: 3,357.7–3,473.8), and 3,351.0 (95% CI: 3271.5–3432.3), respectively (*P* for trend: 0.031). These results indicated that depression severity was independently correlated with lung function decline in Chinese college students. Future prospective cohort or interventional studies are needed to confirm the negative association between depressive symptoms and lung function and investigate its causality.

## Introduction

Lung function has proved to be an effective and non-invasive measure of the respiratory health of individual patients and populations. Its assessment not only helps to identify people with a potential risk for COPD, but may also predict survival in asymptomatic adults who do not have chronic respiratory diseases or persistent respiratory symptoms (Burney and Hooper, 2011), as well as in people with numerous other adverse health conditions, including metabolic syndrome (Oda and Kawai, 2009), type 2 diabetes mellitus (Yeh et al., 2005), cardiovascular disease (Johnston et al., 2008), and lung cancer (Mannino et al., 2003). Thus, it is important to identify risk factors that negatively affect lung function in order to enable earlier detection of morbidity.

Although the exact mechanisms leading to lung function decline are not entirely clear, oxidative stress may play a crucial role. Oxidative stress occurs as a result of oxidant/antioxidant imbalance (Valko et al., 2007). It induces lipid peroxidation, protein oxidation, and DNA damage, which, in turn, results in the development of abnormalities in cell structures, eventually leading to cell death (Valko et al., 2007). Because the surface area for gas exchange is large, the respiratory system is particularly susceptible to oxidative stress-induced injury, which subsequently exacerbates pulmonary dysfunction (Santus et al., 2014).

Forced vital capacity, a simple and accurate measure of lung function, is one of the most important indexes for assessing vital capacity in adults (Chhabra, 1998) and determining the total lung capacity (Vandevoorde et al., 2008). In addition, evidence indicates that FVC predicts survival in asymptomatic adults without chronic respiratory diagnoses or persistent respiratory symptoms (Burney and Hooper, 2011). Therefore, FVC has been chosen as an indicator of lung function (Pellegrino et al., 2005). Furthermore, the results of a case-control study revealed that the level of malondialdehyde, which is a useful marker of exacerbation-associated oxidative stress in patients with COPD, was higher in patients with stable COPD than in healthy controls (Antus et al., 2014).

Similarly, a previous epidemiological study has shown that 36% of patients with COPD reported having depression (Rigual et al., 2017). This may be because oxidative stress is also a well-recognized factor in the pathophysiology of depression. A population-based meta-analysis showed that patients with depression have elevated oxidative stress levels (Black et al., 2015). This can be due to abnormal hypothalamic-pituitary-adrenal axis activity and immuno-inflammatory dysregulation (Penninx et al., 2013). These studies provided powerful evidence that depression is positively associated with lung function decline. However, thus far, it has only been observed that depression could increase the risk for COPD in middle-aged and older adults (Di Marco et al., 2006) and it remains unknown whether it has a negative effect on the lung function in healthy adults. The Global Initiative for Chronic Obstructive Lung Disease has also proposed that future studies should be conducted to evaluate the lung function in a variety of populations exposed to various risk factors, such as cigarette smoking, obesity, and depression (Pauwels et al., 2001).

Therefore, in this study, we aimed to investigate whether depression severity is associated with lung function decline in Chinese college students.

## Materials and Methods

### Ethics Statement

Ethics approval was obtained from the Institutional Review Board of the College of Physical Education of Southwest University. All participants or the parents or legal guardians of those aged < 16 years provided written informed consent.

### Study Design and Participants

This was a cross-sectional study that included college freshmen from 35 schools/colleges of Southwest University between October and December 2018. Participants were recruited by stratified cluster sampling. They were a part of the ongoing Southwest University Physical Fitness and Health cohort study (2018–2021) being conducted to assess the association between physical fitness and the health status of college students in Southwest University, a key national comprehensive university under the direct administration of the Ministry of Education. They were asked to participate in a structured and self-administered health status questionnaire survey. The detailed questionnaire content has been previously published (Ren et al., 2020). Only those who provided informed consent were included in this study.

All participants underwent annual physical fitness examinations, including assessment of lung function (FVC) and physical fitness status (50-m sprint, sit and reach, standing long jump, 800/1,000-m run, and sit-ups/pull-ups), at the physical fitness examination center of Southwest University. Participants with missing data were excluded.

### Assessment of Lung Function

Lung function was assessed by measuring the FVC using a spirometer (CSTF-FH, Tsinghua Tongfang). All participants were requested to hold the spirometer and perform forced expiration in a standing and stationary position. Each participant was asked to make two attempts, and the higher value of the two measurements was recorded as the FVC.

### Assessment of Depression Severity

A validated Chinese version of the Zung SDS was used to assess the severity of depression (Peng et al., 2013). The SDS has 20 items and the score of each item ranges from one to four, with a sum score ranging from 20 to 80. A higher SDS score indicates a greater depression severity. In the current study, SDS scores of ≥40 and ≥45 indicated mild and moderate-to-severe depression, respectively. Accordingly, participants were divided into three groups: normal, mild depression, and moderate-to-severe depression.

Additionally, the SDS also assesses anxiety symptoms via the following five questions: “Do you feel down-hearted and blue?” (Chan et al., 2010), “Do you have trouble sleeping at night?” (Zung, 1971), “Does your heart beat faster than usual?” (Zung, 1971), “Do you get tired for no reason?” (Zung, 1971), and “Do you still enjoy the things you used to?” (Chan et al., 2010). The scores of these five items are summed as a total score, which ranges from 5 to 20; higher scores indicate greater anxiety severity. In this study, anxiety was defined as a score ≥6. In this study, Cronbach’s α coefficient for the SDS was 0.738, indicating a good internal consistency.

### Relevant Covariates

To control for covariates, we also measured potential relevant covariates, based on a previous study (Ren et al., 2020), including sex, age, BMI, only one child, parent’s educational levels and marital status, smoking and drinking status, sleep duration and quality, breakfast frequency, and PA. The standing long jump was additionally used to assess muscular fitness. All participants stood behind the starting line and were told to push off vigorously and jump as far as possible. They had to land with their feet apart and remain upright. Each participant made two attempts and the greater of the measurements was recorded as the standing long jump.

### Statistical Analysis

Statistical analysis was performed using IBM SPSS Statistics 24.0 software (IBM SPSS Inc., Chicago, IL, United States). All continuous variables were expressed as geometric least square means (95% confidence intervals [CIs]), and categorical variables were expressed as percentages. Because the distributions of all continuous variables were skewed, all continuous variables were log-transformed prior to performing ANCOVA. Differences in the participants’ characteristic among the groups were estimated using ANCOVA for continuous variables and multiple logistic regression analysis for categorical variables, after adjustment for sex (categorical variable) and age (continuous variable).

Forced vital capacity and depression severity were used as dependent and independent variables, respectively. ANCOVA was also conducted to examine the relationship between depression severity and FVC. In model 1, the analysis was conducted without any adjustments. Model 2 was adjusted for sex (categorical variable) and age (continuous variable). Model 3 was further adjusted for the following factors: BMI (continuous variable), one child (categorical variable), father’s education (categorical variable), mother’s education (categorical variable), parent’s marital status (categorical variable), smoking status (categorical variable), drinking status (categorical variable), PA (categorical variable), sleep duration (categorical variable), sleep quality (categorical variable), breakfast frequency (categorical variable), standing long jump (continuous variable), and anxiety symptoms (categorical variable). *P*-values < 0.05 were considered statistically significant for all two-sided tests.

## Results

A total of 4,550 college freshmen were recruited. Among them, 4,258 provided written informed consent. We excluded 367 participants owing to missing data on sleep duration (*n* = 4) and FVC (*n* = 363). Finally, 3,891 participants (1,320 males and 2,571 females) with an age range of 16–24 years (mean 18.1 years, standard deviation 0.7) were included in the study.

Sex- and age-adjusted participants’ characteristics according to the depression severity are shown in Table 1. The proportion of females was lower (*P* for trend: 0.002), whereas that of younger subjects was higher (*P* for trend: 0.004) in the moderate-to-severe depression group. Participants with moderate-to-severe depression reported a higher frequency of occasional drinking and skipping breakfast, had lower PA levels, and lower proportion of good sleep quality (all *P* for trends: ≤0.005). Depression severity was significantly negatively related to standing long jump (*P* for trend: 0.042). There were no significant differences between other participants’ characteristics and depression severity. Distribution of FVC according to different depressive level are also shown in Supplementary Table S1.

**TABLE 1:** Sex- and age-adjusted participants’ characteristics according to depressed level.

***N* = 3891**	**Normal (*n* = 3018)**	**Mild depressed (*n* = 574)**	**Moderate and serious depressed (*n* = 299)**	***P* for trend^1^**
SDS range, score	23–39	40–44	45–60	–
**Demographic characteristics**				
Sex (female)	64.8	72.0	67.9	0.002
Age, year	18.1 (18.1, 18.1)	18.2 (18.1, 18.2)	18.2 (18.2, 18.3)	0.004
**Only one child, %**	50.9	50.7	49.8	0.859
Father education, %				
Senior high school or less	68.1	69.0	69.9	0.646
College	29.3	28.2	28.8	0.876
Mother education, %				
Senior high school or less	74.5	75.1	76.6	0.555
College	24.1	22.8	22.1	0.466
Parent’s marital status, %				
Married	89.1	88.5	89.3	0.980
Widowed	8.5	9.6	8.0	0.871
Divorced	2.5	1.9	2.7	0.810
**Lifestyle factors**				
BMI, kg/m^2^	20.4 (20.3, 20.5)	20.2 (20.0, 20.4)	20.3 (20.0, 20.6)	0.497
Smoking status, %				
Regularly	0.5	1.4	0.7	0.166
Occasionally	2.3	2.3	3.7	0.117
Drinking status, %				
Regularly	0.6	1.0	1.3	0.076
Occasionally	43.6	47.4	48.2	0.005
**Breakfast frequency**				
**≤1 time/week**	1.6	2.1	4.0	0.005
**2–5 times/week**	19.7	33.4	38.5	<0.001
PA, MET⋅h⋅week^–1^ (≥23)	82.7	74.4	67.9	<0.001
Sleep duration(6–8 h), %	90.7	90.2	91.0	0.773
Good sleep quality, %	92.1	73.3	62.9	<0.001
**Anxiety symptoms: “no,” %**	24.4	1.0	1.0	<0.001
**Standing long jump, cm**	185.9 (185.3, 186.4)	185.7 (184.2, 187.2)	183.6 (181.6, 185.7)	0.042

*^1^*P* for trends were assessed using multivariate logistic regression analyses. ^2^ Continuous variables were log-transformed and are expressed as the estimated geometric means (95% confidence intervals).*

Among the 3,891 participants, 299 (7.7%) had moderate-to-severe depression. ANCOVA revealed a significant negative relationship between depression severity and FVC after adjusting for potential confounders. The geometric means of the FVC levels for the normal, mild depression, and moderate-to-severe depression groups were 3,446.1 (95% CI: 3,418.6–3470.3), 3,415.2 (95% CI: 3,357.7–3,473.8), and 3,351.0 (95% CI: 3,271.5–3,432.3), respectively (*P* for trend: 0.031) (Table 2).

**TABLE 2:** Adjusted relationships between depressed level and the FVC.

***N* = 3891**	**Model 1^a^**	**Model 2^b^**	**Model 3^c^**	**Model 4^d^**
**FVC, ml**				
Normal (*n* = 3018)	3,465.2 (3,431.5, 3,499.2)^e^	3,449.6 (3,422.4, 3,473.6)	3,446.1 (3,418.6, 3,470.3)	3,446.1 (3,418.6, 3,470.3)
Mild depressed (*n* = 574)	3,333.8 (3,260.0, 3,409.4)	3,408.4 (3,351.8, 3,467.8)	3,415.2 (3,357.7, 3473.8)	3,415.2 (3,357.7, 3,473.8)
Moderate and serious depressed (*n* = 299)	3,305.0 (3,204.0, 3,409.2)	3,330.9 (3,252.3, 3,409.2)	3,340.9 (3,261.7, 3,422.1)	3,351.0 (3,271.5, 3,432.3)
P for trend^f^	0.004	0.006	0.019	0.031

*FVC = forced vital capacity; BMI = body mass index; PA = physical activity; MET = metabolic equivalents; ANCOVA = analysis of covariance. ^*a*^Model 1: Crude. ^*b*^Model 2: Adjusted for sex, age (continuous variable). ^*c*^Model 3: Additionally adjusted for BMI (continuous variable), only on child (yes or no), father education (senior high school or less, college or undergraduate), mother education (senior high school or less, college, or undergraduate), parent’s marital status (married, widowed, divorced), smoking status (regularly, occasionally, never), drinking status (regularly, occasionally, never), PA (≥23 MET⋅h⋅week-1 or not), sleep duration (6–8 h or not), good sleep quality (yes or no), anxious symptoms (yes or no), and breakfast frequency (≤1 time/week, 2–5 times/week, and ≥6 times/week). ^*d*^Model 4: Additionally adjusted for standing long jump (continuous variable). ^*e*^Adjusted data are expressed as estimated geometric means (95% confidence intervals). ^*f*^*P* for trend were obtained using ANCOVA.*

## Discussion

In this cross-sectional study, we examined the relationship between depression severity and FVC in Chinese college students. ANCOVA showed that moderate-to-severe depression was significantly and independently associated with a reduced FVC, after adjustment for potential confounders.

A meta-analysis, which included 16 prospective cohort studies and 28,759 individuals aged ≥ 42 years, demonstrated a positive association between depression and increased risk for COPD (Atlantis et al., 2013). However, it is noteworthy that the participants of those studies were limited to elderly patients with COPD. The present is the first study to examine the relationship between depression and lung function, assessed by FVC, among Chinese healthy adults.

Although the exact mechanism underlying the debilitating effect of depression on lung function remains unclear, it may be explained by the role played by the immune system. It is widely known that overproduction of pro-inflammatory cytokines in the body may cause deterioration of multiple subsequent immunological functions by elevating the levels of plasma adrenocorticotropic hormone, which is followed by an increase in cortisol levels, resulting in depression (Kiecolt-Glaser and Glaser, 2002). Additionally, it is known that depression could enhance the production of pro-inflammatory cytokines (Kiecolt-Glaser and Glaser, 2002). Interestingly, the overproduction of pro-inflammatory cytokines also induces abnormal endothelial function, leading to impaired lung alveolar function and consequent persistent lung function decline (Jiang et al., 2008).

Unhealthy dietary behaviors could also explain our findings. A previous study showed that the consumption of energy-dense foods was higher in individuals with depression than in healthy individuals (Payne et al., 2012). Consumption of such foods could result in systemic inflammation (Azadbakht et al., 2017), which directly induces the innate immune response via the activation of Toll-like receptor 4 by circulating free fatty acids. This may induce increased production of pro-inflammatory cytokines, such as interleukin-6 (IL-6), tumor necrosis factor alpha, and C-reactive protein. Of these, IL-6 is a powerful inducible factor of neutrophil responses, which could cause airflow obstruction (Shaw et al., 2007), subsequently contributing to lung function decline.

Finally, oxidative stress may also mediate this relationship. Depression is characterized by activated oxygen and nitrogen species pathways, which leads to lipid peroxidation, protein oxidation, and DNA damage (Maes et al., 2011; Moylan et al., 2014). Additionally, oxidative stress plays an important role in the development and progression of lung function decline due to lipid peroxidation, protein oxidation, and DNA damage, which, in turn, results in the development of abnormalities in cellular structures, eventually leading to cell death (Valko et al., 2007). The large surface area for gas exchange makes the respiratory system particularly susceptible to oxidative stress-mediated injury, which subsequently exacerbates pulmonary dysfunction (Santus et al., 2014). Unfortunately, we did not investigate pro-inflammatory cytokines, consumption of energy-dense foods, or biomarkers of oxidative stress. Thus, further studies are warranted to examine the relationship of these factors with depression and lung function. The inverse association observed between depression severity and lung function may also provide new insights into the role of depression in the lung function decline related to chronic pain, because severe depression was also present in individuals with COPD experiencing pain (Lee et al., 2017). Further studies should assess the possible association of depression severity with pain and lung function.

The limitations of this study are as follows. First, it was a cross-sectional study; therefore, the causal relationship between depression severity and lung function cannot be determined. Second, although two cut-off points (SDS scores 40 and 45) were used to define subjects having mild and moderate-to-severe depression, we were unable to accurately diagnose depression. Third, our participants were limited to regional Chinese adolescents aged 16–24 years. Hence, our regional results may not be representative of all Chinese college students. Therefore, further studies on other college students are essential to confirm our results. Fourth, pro-inflammatory cytokines and energy-dense foods were not assessed. Whether these factors play important roles as mediators between depression severity and lung function in our population is unknown. As breakfast skippers consume more energy-dense foods (Utter et al., 2007), although additional adjustment for breakfast frequency (Model 3) attenuated this relationship, the negative relationship was confirmed (*P* for trend: 0.031). Fifth, we only considered the influence of depression severity on lung function. The possible associations between other physical functions (50-m sprint, sit and reach, 800/1,000-m run, and sit-ups/pull-ups) and depression remain unknown. Because muscular fitness, but not other physical function components (50-m sprint, sit and reach, 800/1,000-m run, and sit-ups/pull-ups), is associated with both depression severity (Suija et al., 2013) and lung function (Smith et al., 2018), we further adjusted for standing long jump (index of muscular fitness), but the inverse association between depression severity and lung function remained. Therefore, in this study, muscular fitness did not confound the association between depression severity and lung function.

## Conclusion

Our cross-sectional study demonstrated that moderate-to-severe depression was significantly and independently correlated with lung function decline in Chinese college students. Future prospective cohort or interventional studies are warranted to assess the causality of the effects of depression severity on lung function in healthy adults.

## Data Availability Statement

The datasets generated for this study are available on request to the corresponding author.

## Ethics Statement

The studies involving human participants were reviewed and approved by the Institutional Review Board of the College of Physical Education of Southwest University. Written informed consent to participate in this study was provided by the participants’ legal guardian/next of kin.

## Author Contributions

CC and ZR conceived and designed the experiments. LG, JC, PC, DS, BC, NS, MY, YW, SL, CZ, RL and GY performed the experiments and conducted data collection. LG and JC analyzed the data and wrote the manuscript. CC and ZR contributed to the reagents, materials, and analysis tools. LG, JC, CC, ZR, BC, CB and PC revised the manuscript.

## Conflict of Interest

The authors declare that the research was conducted in the absence of any commercial or financial relationships that could be construed as a potential conflict of interest.

## Abbreviations

ANCOVA: analysis of covariance
BMI: body mass index
COPD: chronic obstructive pulmonary disease
FVC: forced vital capacity
IPAQ: international physical activity questionnaire
MET: metabolic equivalents
PA: physical activity
SDS: self-rating depression scale.
