# Supplementary material for: Moderate-to-Severe Depression Adversely Affects Lung Function in Chinese College Students
Source: Front Psychol. 2020 Apr 15;11:652. doi: 10.3389/fpsyg.2020.00652 (PMC7174779; doi:10.3389/fpsyg.2020.00652)
Supplement: Supplementary file 1 [file Table_1.docx]

**Table S1 Distribution of FVC according to different depressive level**

| **N =3891** | **FVC** | | | | | |
| --- | --- | --- | --- | --- | --- | --- |
|  | **median (IQR), ml** | **the distribution of FVC percentile** | | | | |
|  |  | **10th** | **25th** | **50th** | **75th** | **90th** |
| **Depressive level** |  |  |  |  |  |  |
| **Normal (n = 3018)** | **3403.5 (2870.8, 4210.3)** | **2475.0** | **2870.8** | **3403.5** | **4210.3** | **5001.0** |
| **Mild depressed (n = 574)** | **3254.5 (2800.0, 3974.5)** | **2400.0** | **2800.0** | **3254.5** | **3974.5** | **4753.5** |
| **Moderate and serious depressed (n = 299)** | **3231.0 (2800.0, 3935.0)** | **2441.0** | **2800.0** | **3231.0** | **3935.0** | **4652.0** |

**IQR: interquartile range; FVC: forced vital capacity**
